# A Review of the Current Approach and Treatment Landscape for Stage III Non-Small Cell Lung Cancer

**DOI:** 10.3390/jcm13092633

**Published:** 2024-04-30

**Authors:** Arthi Sridhar, Hina Khan, Binoy Yohannan, Kok Hoe Chan, Nilansh Kataria, Syed Hasan Jafri

**Affiliations:** 1 Department of Oncology, Mayo Clinic, Rochester, MN 55901, USA; 2 Division of Hematology and Oncology, Department of Internal Medicine, University of Texas Health Sciences Center at Houston, Houston, TX 77030, USA; 3 Department of Hematology, Mayo Clinic, Rochester, MN 55901, USA; 4 Department of Internal Medicine, MedStar Washington Hospital Center, Washington, DC 20010, USA; nilansh.kataria@medstar.net

**Keywords:** precision, oncology, personalized, PD-L1, immunotherapy, targeted, osimertinib, durvalumab, radiation, chemotherapy

## Abstract

The therapeutic landscape of the management of stage III non-small cell lung cancer (NSCLC) has drastically evolved with the incorporation of immunotherapy and targeted therapy. Stage III NSCLC accounts for one-third of the cases and the treatment strategy of these locally advanced presentations are diverse, ranging from surgical to non-surgical options; with the incorporation of chemo-immunotherapy, radiation, and targeted therapies wherever applicable. The staging of this disease has also changed, and it is essential to have a strong multidisciplinary approach to do justice to patient care. In this article, we aim to navigate the nuanced approaches in the diagnosis and treatment of stage III NSCLC and expand on the evolution of the management of this disease.

## 1. Background

Lung cancer is the second most common cancer worldwide and one of the deadliest malignancies associated with tobacco smoking. In the United States, lung cancer is the principal cause of cancer-associated deaths in both sexes, with an estimated 238,340 new cases and 127,070 deaths predicted in 2023 [1]. More people die from lung cancer than from breast, brain, colorectal, and prostate cancers combined [2]. Research into tumor biology, early diagnosis, and biomarker-driven precision oncology has led to decreased lung cancer mortality [3]. In addition, increased awareness amongst the public has led to a significant reduction in worldwide smoking rates, thereby lowering the lung cancer incidence [4].

Lung cancer is a heterogenous disease; most cases (85%) are non-small cell lung cancer (NSCLC), with adenocarcinoma being the most common histology [5]. Early-stage NSCLC (I–II) accounts for only 16% of cases and is usually defined as tumors ≤7 cm with no regional lymph node involvement (N0) and without invasion into adjacent vital structures or tumors ≤5 cm with N1 node involvement [6]. Locally advanced NSCLC (LA-NSCLC), or stage III disease, accounts for 25–30% of new cases every year and is characterized by tumors that are >7 cm in size; or tumors >5 cm with N1, N2, or N3 nodes, or invading vital organs [6,7,8]. Stage III lung cancer is a diverse group comprising stage IIIA, IIIB, and IIIC classification based on the eighth edition of the TNM (tumor size, nodal spread, metastasis) lung cancer staging system. Survival also varies, with a 5-year overall survival for patients with pathological stage IIIA disease of 41% versus only 12% in those with stage IIIC disease [9]. The clinical management of stage III disease also varies widely, with the sequence and choice of multimodality therapy depending heavily on individual and institutional preference. Some aspects of the controversies in managing stage III NSCLC arise from the major limitations of previous studies, such as small sample size, heterogenous patient populations, non-randomized data, inadequate power, and short follow-up. In addition, the definition of stage III disease has evolved with time. This lack of standardization has created several challenges in improving outcomes.

The mainstay of treatment for LA-NSCLC is definitive local therapy (surgery or radiation) combined with a platinum-based doublet chemotherapy. Surgery is the preferred therapeutic option in some patients with stage IIIA disease [10]. The decision about which local therapy modality to use (surgery vs. radiation) is based on medical comorbidities, performance status, and disease burden in the mediastinal nodes. Adjuvant chemotherapy can improve 5-year overall survival by 4–15% in patients with completely resected NSCLC [11,12,13].

Historically, for patients with inoperable stage III disease, concurrent chemotherapy and radiation is the preferred treatment approach, and this modality has shown better response and survival than sequential therapy [14]. Outcomes were poor for patients with stage III disease prior to the era of immunotherapy, with a 3-year OS benefit of concurrent chemoradiation for stages IIIA/B of 23.8% [6]. The addition of higher doses of radiation of 75 Gy (RTOG 0617) did not improve outcomes compared to 60 Gy [8]. However, the addition of immune checkpoint inhibitors (ICIs) to chemotherapy has changed the therapeutic landscape for stage IV NSCLC, and ICIs are currently also approved for earlier-stage NSCLC. PACIFIC trial data showed a significant improvement in progression-free survival (PFS) and overall survival (OS) with durvalumab in patients with inoperable stage III disease treated with concurrent chemoradiation [15]. A real-world study also showed that consolidation with immunotherapy led to better survival even in patients treated outside the PACIFIC trial protocol [16]. In this article, we provide an overview of the treatment of stage III NSCLC, particularly focusing on the recent advancements in the last 5 years.

## 2. Staging for Locally Advanced NSCLC

The TNM staging system is based on tumor size, lymph node status, and evidence or absence of metastatic disease. The eighth edition of TNM staging came into effect on 1 January 2018, and its prognostic utility was validated by retrospectively reviewing approximately 95,000 patients. The mutational profile of the tumor is not incorporated into the TNM system. The eighth edition of TNM staging also further refined the stages by increasing the number of stage subcategories [17].

The definition of stage III disease changed with this revised eighth edition. Under the previous staging criteria, stage III was defined as T3 or T4 disease or positive lymph nodes in the mediastinum (N2 or N3) without metastatic disease (M0). In the eighth edition, stage III NSCLC includes tumors >5 cm with involvement of the hilar or peribronchial lymph node (T3N1) and tumors >7 cm (T4), regardless of lymph node involvement. In the seventh edition of TNM, T3N1 tumors were also considered stage III, but the tumor size cutoff was 7 cm, and tumors ≤7 cm without lymph node involvement were categorized as stage IIB. In addition, ipsilateral N2 disease is now staged as IIIB rather than IIIA. Stage IIIC disease was added in the eighth edition for patients with T3/T4 N3 disease, which also reflects the poorer outcomes in this category compared with patients who have stage IIIB cancers [18]. Table 1 shows the eighth edition lung cancer staging paradigm [18].

## 3. Imaging Staging Modalities

The TNM staging system is based on tumor size, lymph node status, and evidence or absence of metastatic disease. The eighth edition of TNM staging came into effect on 1 January 2018; and its prognostic utility was validated by retrospectively reviewing approximately 77,000 patients [17]. The mutational profile of the tumor is not incorporated into the TNM system. The eighth edition of TNM staging also further refined the stages by increasing the number of stage subcategories [17].

Accurate staging prior to establishing a treatment plan is critical to improving outcomes for stage III NSCLC. Chest computed tomography (CT) with intravenous contrast is the preferred imaging modality in all patients with suspected lung cancer. Contrast-enhanced studies improve the evaluation of lymph nodes, pleura, and pericardial involvement, as well as assessment for metastatic disease [19].

However, CT scan with contrast alone is not enough. A significant proportion (up to 24%) of patients with stage III NSCLC who have a negative CT scan may have evidence of occult extra-thoracic metastatic disease on PET-CT [20]. Moreover, PET-CT, when combined with conventional imaging work-up, significantly reduces unnecessary thoracotomies [21]. It is also not uncommon to have false-negative PET-CT results, especially in patients with tuberculoma and inflammatory pseudotumor [22,23]. Hence, FDG-avid lesions seen on PET-CT should be biopsied and confirmed pathologically. False-negative results are problematic, as 7–16% of patients with NSCLC may have occult lymph node metastasis despite a negative FDG-PET/CT [24,25].

PET-CT combines anatomic and metabolic data and has a sensitivity of 58–94%, and a specificity of 76–96% for detection of disease in mediastinal lymph nodes [26]. A Cochrane Review from 2014 showed that PET-CT scanning (SUV_max_ ≥ 2.5) has a sensitivity of 81.3% (95% CI, 70.2–88.9) and a specificity of 79.4% (95% CI, 70–86.5). The sensitivity for detection of lymph nodes on PET-CT scan (defined as SUV_max_ > mediastinal background) was 77.4% (95% CI, 65.3–86.1), and specificity was 90.1% (95% CI, 85.3–93.5); however, the pathologic evaluation of lymph nodes is still standard of care [27]. It is also important to remember that FDG-PET is not sensitive enough to image tumors ≤ 2 cm. In addition, false-negative PET-CT results can also be seen with lepidic carcinoma, bronchoalveolar carcinoma, and well-differentiated adenocarcinoma [28].

Approximately 20% of stage III lung cancer patients may have occult central nervous system (CNS) metastasis, and a PET-CT scan is not adequate to rule out CNS metastases [29,30]. Dedicated CNS imaging, preferably MRI with gadolinium contrast, is preferred to rule out occult brain metastasis.

## 4. Pathologic Mediastinal Staging

Stage III NSCLC patients being treated with curative intent should undergo a pathologic assessment of the suspicious mediastinal lymph nodes, as imaging alone is inadequate. When feasible, biopsy samples should be obtained from a target lesion that would help establish the highest stage. Lymph node assessment can be done either via minimally invasive endosonography or mediastinoscopy [31].

**Figure 1:**
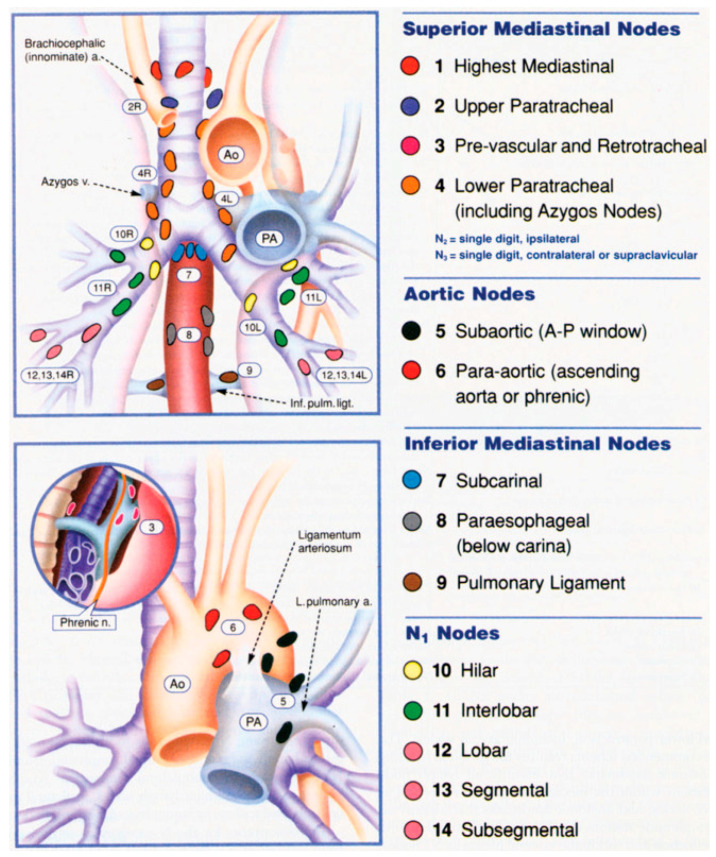
This figure highlights the regional lymph node stations for lung cancer staging. Lung cancer is staged based on the level of lymph node involvement and the side of the lymph nodes involved in relation to the primary tumor (from Mountain CF, Dresler CM. Chest 1997;11:1718–23 [34]).

Endosonography using either endobronchial ultrasound (EBUS) and/or endoscopic ultrasound (EUS) are viable minimally invasive alternatives to mediastinoscopy. EBUS provides adequate access to hilar, mediastinal, and interlobar lymph nodes; however, it is not a good option for perivascular, aortopulmonary, para-aortic, pulmonary ligament, and paraesophageal nodes (Figure 1: lymph node stations 3a, 5, 6, 8, and 9) [32]. In patients with an abnormal mediastinum on imaging, the sensitivity of EBUS alone is 94–95% [32,33].

The sensitivity and specificity of EUS are 83% and 89% respectively [27,30,31]. EUS offers access to lymph node stations 4L, 5, 7, 8, and 9, which are not normally accessible by mediastinoscopy or EBUS. Nodes that are anterolateral to the trachea (stations 2R, 2L, 4R) are difficult to sample reliably (but are commonly involved in lung cancer) [35].

Cervical mediastinoscopy under general anesthesia is an important diagnostic modality for pathologic assessment of the superior mediastinum. The accuracy of cervical mediastinoscopy depends on the number of biopsies and number of lymph node stations evaluated. Cervical mediastinoscopy can biopsy lymph nodes in stations 2R, 2L, 4R, 4L, and 7. It has a sensitivity of 78% and a negative predictive value of 91% [35,36].

Patients with enlarged hilar nodes seen on CT and/or FDG PET/CT (N1 disease) should undergo invasive mediastinal staging even if the mediastinal nodal involvement is negative on imaging [24,37].

A proportion of patients who undergo resection will have mediastinal lymph node metastatic disease that is missed on current imaging techniques [38]. Hence, ACCP guidelines recommend an invasive endosonographic mediastinal evaluation for this category of patients [39]. In addition, European Society of Thoracic Surgeons (ESTS) guidelines suggest endosonographic staging in patients with suspicious hilar lymph nodes on imaging, peripheral tumors (>/=3 cm), and FDG-non-avid primary tumors. Similarly, endosonographic mediastinal exploration is recommended by ESTS in patients with central lung tumors (negative hilar and mediastinal lymph nodes on imaging) due to the high false-negative rates of CT (20–25%) and FDG PET-CT (24–83%) [40].

However, if endosonography-guided biopsy of N2 or N3 nodes is negative, a mediastinoscopy can be performed to exclude a false-negative result [36]. The role of confirmatory mediastinoscopy in patients with a negative EBUS/EUS is still under debate and is currently being investigated in a multicenter, randomized controlled trial (MEDIASTrial) [41].

## 5. Treatment Approaches Prior to Checkpoint Inhibitor Trials

### Chemoradiotherapy

For unresectable stage III NSCLC, the historical standard of care prior to the introduction of immunotherapy and targeted agents was concurrent chemoradiation therapy. Randomized trials established the survival benefit of induction chemotherapy followed by radiation compared to radiation therapy alone [42,43,44]. In a trial by Dilman et al., 155 patients with stage III NSCLC were randomized to receive radiation plus cisplatin-based chemotherapy (group 1) or radiation alone (group 2). Improvement in short- and long-term survival rates was observed (survival rate at 1 year, 55% vs. 40%; and at 3 years, 23% vs. 11%, respectively, in group 1 vs. 2) [43]. In the RTOG 88-08 study, patients with unresectable stage II, IIIA, or IIIB NSCLC (per AJCC TNM 5th edition) were randomized to receive standard fractionated radiation therapy, induction chemotherapy followed by standard fractionation, or a hyperfractionated radiation schedule. The combination chemotherapy plus radiation arm was statistically superior in OS at 1 year compared to the other two arms, with a 1-year survival of 13.8 months compared to 11.4 months in the standard fractionation arm and 12.3 months in the hyperfractionated arm (*p* = 0.03) [43]. The Non-Small Cell Lung Cancer Collaborative Group meta-analysis in 1995 included 52 randomized clinical trials with data from 9387 patients. There was a benefit in OS seen by the addition of cisplatin-based chemotherapy to radical radiotherapy (hazard ratio [HR], 0.87), the addition of cisplatin-based chemotherapy to surgery (HR, 0.87), and the addition of cisplatin-based chemotherapy to best supportive care (HR, 0.73). The greatest benefit was observed from cisplatin-based chemotherapy regimens, whereas alkylator-based regimens showed a detriment [45]. This established the role of cisplatin-based chemotherapy in the management of locally advanced NSCLC.

The benefit of concurrent chemoradiation was further established by a meta-analysis in 2010 conducted by the Non-Small Cell Lung Cancer Collaborative Group, which compared concurrent chemoradiation with sequential chemoradiotherapy [14]. Six pooled randomized controlled trials were included for a total of 1205 patients with a median follow-up of 6 years. Concurrent chemoradiation showed a significant OS benefit over sequential chemoradiation with an HR of 0.84 (95% CI, 0.74–0.95; *p* = 0.004); and at 3 years, an absolute benefit of 5.7%. PFS was also improved by concomitant chemoradiation with an HR of 0.90 (95% CI, 0.79–1.01; *p* = 0.07) and an absolute benefit of 2.9% at 3 years. There was also a benefit in locoregional control from concurrent chemoradiation, with an HR of 0.77 (95% CI, 0.62–0.95; *p* = 0.01), although there was no improvement in the rate of distant progression. The benefit in OS was attributed to better locoregional control, as there was no difference in the rate of distant progression between the two arms. With regard to safety, there was a significantly higher rate of grade 3–4 esophageal toxicity noted in the concomitant chemoradiotherapy arm compared with the sequential treatment arm: 4% versus 18% (relative risk 4.9; 95% CI, 3.1–7.8; *p* < 0.001). There was no significant difference noted in the rates of acute pulmonary toxicity [14]. The improvement in OS in the group receiving concurrent chemoradiation was thought to outweigh the risk of esophageal toxicity. Thus, this established concurrent chemoradiotherapy as the standard of care for patients with locally advanced NSCLC over sequential chemoradiation.

Chemotherapy regimens commonly used with concurrent radiation are weekly carboplatin with paclitaxel and cisplatin with etoposide. Carboplatin with paclitaxel was studied in a phase 2 trial by Belani et al. in 276 patients with unresectable stage IIIA and IIIB NSCLC [46]. Patients were randomized into three treatment arms. Arm 1 received sequential treatment with paclitaxel 200 mg/m^2^ and carboplatin (AUC = 6) for 2 cycles followed by sequential thoracic radiation therapy (TRT). Arm 2 received induction chemotherapy with paclitaxel 200 mg/m^2^ and carboplatin (AUC = 6) for two cycles followed by weekly paclitaxel 45 mg/m^2^ and carboplatin (AUC = 2) followed by concurrent chemoradiation with TRT. Arm 3 received concurrent chemoradiation with weekly paclitaxel 45 mg/m^2^ and carboplatin (AUC = 2) with TRT followed by consolidation therapy consisting of two cycles of paclitaxel 200 mg/m^2^ and carboplatin (AUC = 6). After a median follow-up of approximately 40 months, median OS was 13.0 months for Arm 1, 12.7 months for Arm 2, and 16.3 months for Arm 3, with the concurrent/consolidative approach seeming to provide the best efficacy results. Treatment-related toxicities during and after radiation therapy were higher in Arm 3, including esophagitis, myelosuppression, and lung toxicities.

Cisplatin plus etoposide with concurrent TRT was investigated in the phase 2 SWOG-9019 trial. Fifty patients with stage IIIB NSCLC were enrolled to receive cisplatin and etoposide with concurrent once-daily RT [42]. The median OS was 15 months (95% CI, 10–22 months) at a median follow-up of 52 months. The 1-, 2-, 3-, and 5-year OS was 58%, 33%, 17%, and 15%, respectively.

For non-squamous histology, another choice of concurrent chemotherapy is cisplatin with pemetrexed, which was based on the phase 3 PROCLAIM trial involving 598 patients with stage IIIA or IIIB unresectable non-squamous NSCLC [47]. Patients were randomly assigned in a 1:1 ratio to receive cisplatin 75 mg/m^2^ and pemetrexed 500 mg/m^2^ IV every 3 weeks with RT for 3 cycles followed by pemetrexed consolidation every 3 weeks for 4 cycles or standard therapy with cisplatin 50 mg/m^2^ and etoposide 50 mg/m^2^ IV every 4 weeks with RT for 2 cycles followed by consolidation platinum-based doublet therapy for 2 cycles. Similar outcomes were noted in both arms, with a median OS of 26.8 months in the cisplatin-pemetrexed arm versus 25 months in the cisplatin-etoposide arm. Grade 3 and 4 treatment-related adverse events were noted less frequently in the cisplatin and pemetrexed arm than in the cisplatin and etoposide arm (64% vs. 76.8%, *p* = 0.001), including neutropenia. This trial was designed to demonstrate the superiority of cisplatin with pemetrexed as compared to cisplatin with etoposide. The trial was terminated early because superiority could not be demonstrated; however, based on the incidence of grade 3 and 4 adverse events, cisplatin with pemetrexed is better tolerated than cisplatin with etoposide [47].

The role of consolidation chemotherapy after chemoradiotherapy has been controversial based on the results from SWOG 9504, and from Hoosier Oncology and U.S. Oncology. Consolidation chemotherapy after chemoradiotherapy initially demonstrated promising results in the SWOG 9504 study [48,49]. This was further investigated in a phase 3 study by the Hoosier Oncology Group and U.S. Oncology in patients with stage IIIA or IIIB NSCLC [50]. Patients were treated with cisplatin and etoposide concurrently with chest radiation. If patients did not have disease progression, they were randomized to receive docetaxel 75 mg/m^2^ IV every 21 days for 3 cycles versus observation. A total of 203 patients were enrolled; 39.4% had stage IIIA disease, and 60.6% had stage IIIB disease. Of the 203 patients, 147 did not progress, 73 patients received docetaxel, and 74 underwent observation. The study failed to meet its primary endpoint of OS (median OS was 21.7 months in the docetaxel arm vs. 23.2 months in the observation arm; *p* = 0.883). Additionally, there were more adverse events in the docetaxel arm, including febrile neutropenia and grade 3–5 pneumonitis; 28.8% of patients in the docetaxel arm required hospitalization, compared to 8.1% in the observation arm, and 5.5% of deaths were attributed to docetaxel. Thus, consolidation with docetaxel did not improve outcomes and increased toxicity, so the study was terminated early [50].

The standard fractionation dose used with concurrent chemotherapy is 60 Gy in 30 daily fractions. A higher dose of radiation (74 Gy) was studied in the phase 3 RTOG 0617 study [8]. Patients were randomized in a 1:1:1:1 design to receive a standard radiation dose of 60 Gy in 30 daily fractions, high-dose radiation of 74 Gy in 37 daily fractions, standard-dose radiation of 60 Gy plus cetuximab, or high-dose radiation of 75 Gy plus cetuximab. All patients received concurrent chemotherapy with weekly carboplatin and paclitaxel, and two cycles of consolidation carboplatin and paclitaxel. Poorer survival was noted in the higher-intensity radiation group (20.2 vs. 28.7 months; HR, 1.38; 95% CI, 1.09–1.76; *p* = 0.004), which was attributed to the higher radiation doses to the heart. There was no difference in median OS noted in patients who received cetuximab versus those who did not (25.0 vs. 24.0 months, respectively), but there was a higher rate of grade ≥3 adverse effects in the cetuximab arm. This study established standard-dose radiation with concurrent chemotherapy excluding cetuximab as the standard of care [8].

Intensity-modulated radiation therapy (IMRT) is preferred to three-dimensional conformal external beam radiation therapy (3D-CRT). This was demonstrated in a secondary analysis of the RTOG 0617 study, which showed that IMRT was associated with a decreased risk of grade ≥3 pneumonitis (7.9% vs. 3.5%; *p* = 0.039) and lower radiation doses to the heart than 3D-CRT [51].

Conventional daily radiation dosing (five daily fractions per week) has been compared to a modified radiation dosing schedule. A meta-analysis of 10 trials with >2000 patients demonstrated that modified fractionated schedules, including accelerated or hyper-fractionated radiotherapy, resulted in significantly higher esophageal toxicities with only a small absolute benefit in OS of 2.5% at 5 years; thus, a daily fraction schedule remains the standard of care in locally advanced NSCLC [52].

## 6. Surgery for Stage III NSCLC

Prior to the introduction of immunotherapy in thoracic oncology, the role of surgery for N2 disease in patients with ipsilateral mediastinal and/or subcarinal lymph node involvement remained unclear. This is likely due to the lack of universal consensus, disease heterogeneity, availability of surgical expertise, and comparable outcomes with or without surgical approaches [52].

In patients with N2 disease, the use of neoadjuvant chemoradiation or chemotherapy before the surgery was compared to chemoradiation alone and showed no survival benefits [53,54,55]. A meta-analysis involving seven randomized trials confirmed no survival benefits with the addition of surgery to chemoradiation in patients with stage III NSCLC [56]. Based on this meta-analysis, treatment-associated mortality was higher in the surgical arms [risk ratio = 3.56 (95% CI: 1.65–7.72), *p* = 0.0005]. An exception to this is patients with tumors in the superior sulcus with T3 or T4, N0 or N1 disease, in whom induction chemoradiation prior to surgery was shown to be feasible to achieve high rates of pathologic complete response and better OS than concurrent chemoradiation [57].

In selected patients such as those with potentially resectable cT1-2, N2 disease (stage IIIA), the introduction of multimodal management, which includes neoadjuvant chemotherapy or neoadjuvant chemoradiation, may downstage the tumor to an expectation of R0 resection. The consensus prior to the era of immunotherapy was to give 2–3 cycles of platinum-based chemotherapy followed by re-evaluation of mediastinal lymph node staging. If the patient responded to downstaging, surgery could be performed for curative intent using complete resection with lymphadenectomy, followed by adjuvant chemotherapy or chemoradiation [58]. However, this treatment paradigm carries a potential risk of mediastinal soft-tissue fibrosis after neoadjuvant therapy, especially with radiotherapy, which may complicate the mediastinal and hilar lymph node dissections.

## 7. Treatment Approach after Checkpoint Inhibitor Trials

### Chemoradiation Followed by Immunotherapy

The introduction of immunotherapy has revolutionized and transformed the treatment landscape for patients with stage III NSCLC. Durvalumab, a humanized IgG1 monoclonal antibody targeting PD-L1, was studied in the phase 3 randomized PACIFIC trial, which compared the use of durvalumab adjuvant versus placebo in eligible patients with locally advanced unresectable stage III NSCLC after definitive concurrent platinum-based chemoradiation. In this trial, the use of consolidative chemotherapy was not allowed, but induction chemotherapy was permitted, and almost 26% of patients received this. Patients were randomly assigned 2:1 after concurrent chemoradiation to receive either durvalumab intravenously at a dose of 10 mg/kg (around 1–42 days after chemoradiation) or placebo every 2 weeks for up to 1 year. The OS in patients who received adjuvant durvalumab maintenance was significantly superior to that of the placebo group (47.5 vs. 29.1 months; stratified HR for death, 0.71; 95% CI, 0.57–0.88) [59]. In a follow-up study after 4 years, approximately 49.6% of patients who received adjuvant durvalumab maintenance remained alive, compared with only 36.3% of patients in the placebo arm. Moreover, among patients who had received durvalumab maintenance and remained alive, 35.3% did not experience progression compared to 19.5% in the placebo arm. Most importantly, durvalumab is tolerable, with similar rates of grade 3/4 adverse events between both groups. Pneumonitis was the most common grade 3/4 toxicity, and was reported in up to 4.4% in the cohort who received durvalumab maintenance versus 3.8% in the placebo cohort, but this was not statistically significant [60]. Due to questionable tolerability of concurrent chemoradiation, the phase 2 PACIFIC-6 trial was designed to evaluate the tolerability of durvalumab given every 4 weeks for up to 24 months after sequential chemoradiation [61]. The study showed a comparable safety profile when compared to the PACIFIC trial. Improved clinical outcomes and immunomodulation with novel immune checkpoint inhibition combinations was explored the phase 2 trial, COAST [62]. The study demonstrated an improved PFS with the combination durvalumab plus monalizumab (anti-NKG2A monoclonal antibody) (HR, 0.42), or durvalumab plus oleclumab (anti-CD73 monoclonal antibody) (HR, 0.44) when compared to durvalumab alone after concurrent chemoradiation. A numerically higher objective response rate was also noted with durvalumab plus oleclumab (30%) and durvalumab plus monalizumab (35.5%) versus durvalumab (17.9%) [62].

Instead of using immunotherapy as a sequential regimen after concurrent chemoradiation, the DETERRED part II trial is a single-institution, phase 2 clinical trial that investigated the feasibility of simultaneous concurrent immunotherapy with intravenous atezolizumab at a dose of 1200 mg given every 3 weeks, and chemoradiation with low-dose carboplatin/paclitaxel, followed by two full cycles of carboplatin/paclitaxel and atezolizumab as consolidation chemotherapy, then atezolizumab maintenance for up to 12 months. There were 30 patients enrolled in the study. At a median follow-up of 39.2 months, median PFS was 15.2 months and median OS was not reached. Patients with targeted mutation and PD-L1 < 1% have lower PFS and OS as compared to those without targetable mutations and PD-L1 ≥ 1%. Grade 3/4 adverse events were observed in 57% of patients. Pneumonia was the most frequent reported adverse event (20%). There were three patients (10%) with radiation pneumonitis: 2 with grade 2 and 1 with grade 3, hence atezolizumab was discontinued [63,64]. Table 2 highlights some major phase 2 and 3 trials in this space.

## 8. Frontline Surgery Followed by Adjuvant Immunotherapy

With the advent of immunotherapy, the role of these agents in the adjuvant setting is also being explored. Two major trials—IMpower 010 (atezolizumab) and KEYNOTE 091 (pembrolizumab)—are evaluating the role of adjuvant ICIs in NSCLC [70,71].

IMpower010 is a phase 3 study evaluating atezolizumab after adjuvant cisplatin-based chemotherapy in patients with completely resected stage IB–IIIA NSCLC (per the AJCC TNM seventh edition staging system) [71]. After randomization, patients received either 1200 mg atezolizumab every 21 days for up to 16 cycles or best supportive care. The primary endpoint for this study was disease-free survival (DFS). The interim analysis, after a median follow-up of 32.2 months in the stage II–IIIA population, demonstrated that the atezolizumab arm had an improved DFS compared with best supportive care in patients with PD-L1 ≥ 1% (HR, 0.66; 95% CI, 0.50–0.88; *p* = 0.0039). In the secondary DFS endpoint analysis, patients with PD-L1 ≥ 50% have an unstratified HR of 0.43; 95% CI, 0.27–0.68); whereas patients with PD-L1 1–49% have an unstratified HR 0.87, 95% CI 0.60–1.26. This highlights that the majority DFS benefit was driven by tumors with PD-L1 ≥ 50%. Based on these results, adjuvant atezolizumab was approved in several countries, including the United States, for patients with stage II–IIIA disease and with PD-L1 ≥ 1% after resection and platinum-based chemotherapy. The first pre-specified interim analysis of OS at a cutoff of 46 months revealed no OS benefit seen with atezolizumab in the intent-to-treat (ITT) population (HR, 0.995; 95% CI, 0.78–1.28) or in the all-randomized population (HR, 0.95; 95% CI, 0.74–1.24). The OS of the PD-L1 < 1% group showed no difference (HR, 1.36; 85% CI, 0.93–1.99). Participants with PD-L1 ≥ 1% demonstrated a trend toward improved OS in the atezolizumab group; however, this was not significant (HR, 0.71; 95% CI, 0.49–1.03). In the PD-L1 > 50% group, an OS benefit was seen with atezolizumab (HR, 0.43; 95% CI, 0.24–0.78). An OS benefit in this biomarker-positive group was seen, irrespective of EFR/ALK mutation status [71].

KEYNOTE 091 is a phase 3 trial studying adjuvant pembrolizumab (pembro) in resected stage IB–IIIA NSCLC (per the AJCC seventh edition) after adjuvant chemotherapy, as indicated [70]. Patients (n = 1177) were randomized 1:1 to receive pembro 200 mg or placebo every 3 weeks for a total of 12 months, or 18 doses. In the final DFS analysis conducted, the median time from randomization to data cutoff was 51.7 months. Here, the DFS was not tested for statistical significance as the success criterion for this endpoint was already met at the second interim analysis. The updated DFS for patients who received adjuvant chemotherapy (median 53.8 vs. 40.5 months; HR 0.80; CI, 0.67–0.96) was found to be consistent with previous data (median 53.6 vs. 42.0 months; HR, 0.76; 95% CI 0.63–0.91; *p* = 0.0014). However, a significant benefit in the tumor propensity score (TPS) ≥ 50% could not be replicated, as seen in prior analysis (median NR in both arms; HR, 0.83; 95% CI, 0.59–1.16, *p* = 0.13). Future DFS and OS data are still pending [72].

## 9. Neo-Adjuvant Chemotherapy and Immunotherapy Followed by Surgery

There is clear advantage of using adjuvant chemotherapy in surgically resectable stage II and IIIA NSCLC, as demonstrated in a meta-analysis that showed a 5-year absolute benefit of adjuvant chemotherapy to be 5.4% [73]. Another meta-analysis evaluated the benefit of neoadjuvant chemotherapy and showed similar results, with an absolute survival improvement of 5% at 5 years [58]. With the advent of immunotherapy, multiple studies have evaluated the use of neoadjuvant PD-1/PD-L1 and CTLA-4 inhibitors and have shown a major pathologic response rate of anywhere between 14% and 45% [74,75,76,77,78,79,80]. This prompted several phase 3 studies to evaluate the incorporation of neoadjuvant immunotherapy. Neoadjuvant immunotherapy was promising, as it had the potential to achieve a maximum pathologic response prior to surgery, and the degree of response could guide management in the adjuvant setting.

CHECKMATE 816 was the first phase 3 study to evaluate neoadjuvant nivolumab plus chemotherapy in patients with resectable stage IB–IIIA non-small cell lung cancer (per the AJCC seventh edition staging system) with no epidermal growth factor receptor (EGFR)/ALK mutations [81]. Patients were randomized to receive three cycles of nivolumab 360 mg every 3 weeks with a platinum doublet, or a platinum doublet alone (n = 358). Patients underwent resection of the tumor 6 weeks after completion of neoadjuvant therapy; and adjuvant chemotherapy, radiation, or both was permitted. Most of the patients in the treatment arm (83.2%) and the control arm (75.4%) underwent surgical resection and 20% and 32%, respectively, received adjuvant therapy. The pCR was 24% in the treatment arm and 2.2% in the control arm (OR, 13.94; 99% CI, 3.49–55.75; *p* < 0.001). At a minimum follow-up of 21 months, the co-primary endpoint, event-free survival (EFS), in the chemotherapy plus immunotherapy arm was 31.6 months, compared with 20.8 months in the chemotherapy-only arm (HR for disease progression, disease recurrence, or death, 0.63; 97.38% CI, 0.43–0.91; *p* = 0.005). When EFS was evaluated by the baseline disease stage, a higher magnitude of benefit was seen in patients with stage IIIA disease (n = 228, median EFS, 31.6 vs. 15.7 months; HR, 0.54, 95% CI, 0.37–0.800). An increasing EFS benefit was seen with increasing PD-L1 expression. The median EFS in patients with PD-L1 < 1% was 25.1 months in the chemotherapy plus immunotherapy arm compared with 18.4 months in the chemotherapy-only arm (HR, 0.85; 95% CI, 0.54–1.32). In patients with PD-L1 ≥ 1%, the median EFS in the chemotherapy plus immunotherapy arm was not reached, compared with 21.1 months in the chemotherapy-only arm (HR, 0.41; 95% CI, 0.24–0.70). In addition, an increased magnitude of benefit in EFS was observed in patients with non-squamous histology (HR 0.50, 95% CI, 0.32–0.79) versus that in patients with squamous histology (HR 0.77, 95% CI, 0.49–1.22) [81].

The NADIM-II trial aimed to evaluate if neoadjuvant nivolumab could be extended to those with Stage IIIB disease and demonstrate a pathological complete response (pCR) [82]. Patients with stage IIIA or IIIB NSCLC were randomized to receive nivolumab plus chemotherapy or chemotherapy alone. Patients with R0 resection received adjuvant nivolumab for one-year [82]. The primary endpoint was pCR by blinded independent pathological review in the ITT population. Neoadjuvant nivolumab was associated with a higher pCR than chemotherapy in the ITT population (36.2% vs. 6.8%; RR, 5.25; 99% CI, 1.32–20.87; *p* = 0.0071). Definitive surgery was achievable for 91% of patients in the treatment arm and 69% of patients in the control arm. In the ITT experimental arm, patients with pCR had higher PD-L1 TPS than non-responders. At a median follow up of 21.9 months, PFS and OS at 24 months was 67.3% (95%CI: 55.5–81.6) and 85.3% (95%CI: 75.7–96.1) in the treatment arm versus 52.6% (95%CI: 36.8–75.2) and 64.8% (95%CI: 47.4–86.4) in the control arm. PD-L1 ≥ 1% was significantly associated with improve PFS (HR: 0.26; 95%CI: 0.08–0.77; *p* = 0.015) [82]. 

The neoSCORE phase 2 trial aimed to answer the question of the optimal number of neoadjuvant cycles of treatment. The trial assessed the efficacy and safety of two vs. three cycles of neoadjuvant sintilimab in combination with platinum-based chemotherapy I resectable IB-IIIA NSCLC [83]. A total of 60 patients were randomized and distributed in a 1:1 ratio to receive either two or three cycles of neoadjuvant therapy. The median follow-up was calculated to be 20.4 months. The major pathological response (MPR) rate was found to be 26.9% (95% CI: 11.6–47.8%) in the two-cycle group as compared to 41.4% (95% CI: 23.5–61.1%; *p* = 0.260) in the three-cycle arm. Notably, a superior MRP rate was seen in squamous NSCLC (51.6%) vs. non-squamous NSCLC (12.5%; *p* = 0.002). To further investigate this, driver mutation next-generation sequencing (NGS) was sent for 95.8% (23/24) of non-squamous NSCLC having sufficient tumor tissue. Mutations such as EGFR, ERBB2, MET, KRAS-G12C, ALK, and RET were detected in 21 patients. Out of the 60 subjects, 55 underwent surgery. Median OS and DFS could not be determined in these patients. The 12-month OS rates in the two-cycle and three-cycle groups were 92.3% and 86.2%, respectively, as compared to the 12- month DFS rates, which were 84.4% and 82.8%, respectively. In terms of safety-related events, 31% of the patients in the two-cycle arm had grade ≥3 treatment-related adverse events (TRAEs) in comparison to 29% in the three-cycle arm [83].

Results from the two-arm phase 2 NEOSTAR trial (n = 44) demonstrated a major pathological response (MPR) of 50% in the neoadjuvant dual checkpoint inhibition (ipilimumab/nivolumab) plus chemotherapy arm, and an MPR of 32.1% in the neoadjuvant nivolumab plus chemotherapy arm. A higher MPR (62.5% vs. 41.2%) was seen in patients without known EGFR/ALK alterations [84]. This is an evolving area of research, with much awaited and pending results from other major phase 3 trials evaluating other immune checkpoint inhibitors in the neoadjuvant setting. Major trials evaluating neoadjuvant immunotherapy in this setting are described in table in Section 10.

## 10. Perioperative Chemoimmunotherapy in Stage III NSCLC

More recently, the role of perioperative chemotherapy and immunotherapy is being explored in stage III NSCLC and is a standard-of-care option for the management of this disease.

KEYNOTE- 671 is a phase 3 trial conducted to evaluate perioperative pembrolizumab in resectable stage II, IIIA, or IIIB (N2 stage) NSCLC [85]. A total of 797 patients were randomly distributed in a 1:1 ratio to receive neoadjuvant pembro 200 mg daily or placebo once every 3 weeks, which was given along with cisplatin-based chemotherapy for four cycles. In both groups this was followed by surgery and adjuvant pembro 200 mg daily or placebo once every 3 weeks for up to 13 cycles. In the first interim analysis, the median follow-up was found to be 25.2 months. There were two primary endpoints calculated at the end of 24 months: event-free survival and overall survival. EFS was higher in the pembro group (62.4% vs. 40.6%; HR for progression, recurrence, or death, 0.58; 95% CI: 0.46–0.72; *p* < 0.001). The results for OS did not fulfil the significance criterion. For secondary endpoints, major pathological response was higher in the pembro group (30.2% vs. 11%; 95% CI, 10.1–18.7; *p* < 0.0001; threshold, *p* = 0.0001), and pathological complete response was observed more in the pembro group as well (18.1% vs. 4.0%; 95% CI, 10.1–18.7; *p* < 0.0001; threshold, *p* = 0.0001). Safety was also measured as a secondary endpoint and 44.9% of the subjects in the pembro group had grade 3 or higher treatment-related adverse events, vs. 37.9% in the placebo group [85].

In addition, AEGEAN is another phase 3 trial which enrolled 802 patients with stage II, IIIA, or IIIB (N2 stage) NSCLC to study the effect of a perioperative durvalumab regimen [86]. Subjects were randomly distributed based on disease stage (II or III) and PD-L1 expression (≥1% or <1%) to receive platinum-based chemotherapy plus durvalumab or placebo every 3 weeks for 4 cycles prior to surgery. This was followed by either adjuvant durvalumab or placebo every 4 weeks for 12 cycles. In the 12-month analysis, the primary endpoint of EFS was found to be higher in the durvalumab group (73.4% vs. 64.5%; stratified HR for progression, recurrence, or death, 0.68; 95% CI: 0.53–0.88; *p* = 0.004). Pathological complete response, another primary endpoint was greater in the durvalumab group as well (17.2% vs. 4.3% at the final analysis; 95% CI, 8.7–17.6; *p* < 0.001 in the interim analysis). Both primary endpoints were observed regardless of disease stage or PD-L1 expression.

Another phase 3 trial, the NEOTORCH study, assessed the effectiveness of toripalimab combined with platinum-based chemotherapy in subjects with stage II or III (N2 stage) resectable NSCLC [87]. A total of 501 patients were randomized equally to receive 240 mg of toripalimab or placebo once every 3 weeks in combination with platinum-based CT for 3 cycles before surgery and 1 cycle after surgery. This was followed by toripalimab 240 mg or placebo once every 3 weeks for up to 13 cycles. In the interim analysis, stage II NSCLC patients were excluded. The median follow-up was estimated to be 18.3 months. Primary outcomes measured included EFS and major pathological response. EFS was not estimable in the toripalimab group compared with 15.1 months in the placebo group (HR, 0.40; 95% CI, 0.28–0.57, *p* < 0.001) and major pathological response was found to be greater in the toripalimab group as well (48.5% vs. 8.4%, between-group difference, 40.2%; 95% CI, 32.2–48.1%, *p* < 0.001). Along the same lines, the secondary outcome of pathological complete response had higher rates in the toripalimab group (24.8% vs. 1.0%, between- group difference, 23.7%; 95% CI, 17.6–29.8%) [87].

This is an evolving area of research and there are numerous challenges including patient selection for neoadjuvant vs. perioperative treatment. There is also need for a predictive and prognostic biomarker that can aid in making therapeutic decisions, and PD-L1 has several limitations as a biomarker [88].

Table 3 highlights current research in this space.

## 11. Targeted Therapies in Stage III NSCLC

The use of targeted therapies in NSCLC has now extended into locally advanced disease. In the phase 3 ADAURA study of 682 patients with stage IB–IIIA NSCLC with an EGFR mutation, patients were randomly assigned to receive adjuvant osimertinib 80 mg daily for up to 3 years versus placebo [95,96]. The primary endpoint of DFS in stage II or IIIA disease showed that 90% of patients in the osimertinib arm and 44% of patients in the placebo arm were alive at a median follow-up of 24 months (overall HR for disease recurrence or death, 0.17; *p* < 0.001). The secondary endpoint of DFS in the overall population showed that, at 24 months, 98% of patients in the osimertinib group and 85% in the placebo group were alive without CNS involvement. OS data is currently premature. This has led to the approval of adjuvant osimertinib in patients with stage II or III resectable locally advanced NSCLC who have an EGFR exon 19 deletion or exon 21 L858R mutation. Osimertinib may also be considered in select patients with stage IB disease and the presence of high-risk features, including lymphovascular invasion, visceral pleural invasion, or a poorly differentiated tumor [97]. Additional studies are ongoing regarding the role of other targeted EGFR agents, as well as the role of adjuvant and neoadjuvant ALK inhibitors in ALK-positive tumors [98,99]. One such study is the phase III ALINA trial studying adjuvant alectinib in comparison to chemotherapy (CT) alone in patients with completely resected stage IB-IIIA (N2 stage) *ALK* positive NSCLC [100]. In this, 257 subjects were randomly distributed in a 1:1 ratio in two groups; one receiving alectinib 600 mg twice daily, and the other getting up to four cycles of platinum-based CT. They were stratified based on stage (IB vs. II vs. IIIA) and race (Asian vs. non-Asian). The major efficacy outcome measures were DFS in the subgroup of patients with stage II-IIIA NSCLC and DFS in the overall study population (stage IB-IIIA). In patients with stage II-IIIA NSCLC, median DFS was not reached in the alectinib arm (95% CI: not estimable [NE], NE) and was 44.4 months in the control arm (95% CI: 27.8, NE) and this was statistically significant (HR 0.24 [95% CI: 0.13, 0.45]; *p* < 0.0001). Similarly significant results were also seen in the overall study population with median DFS not reached (95% CI: NE, NE) in the alectinib arm and 41.3 months (95% CI: 28.5, NE) in the chemotherapy arm (HR 0.24 [95% CI: 0.13, 0.43]; *p* < 0.0001). This led to a recent FDA approval for the use of alectinib in resected NSCLC for patients with an ALK positive (diagnosed by an FDA approved test) NSCLC on April 18, 2024.

## 12. Summary of Treatment Approaches for Stage III NSCLC

### 12.1. Stage IIIA

**Figure 2:**
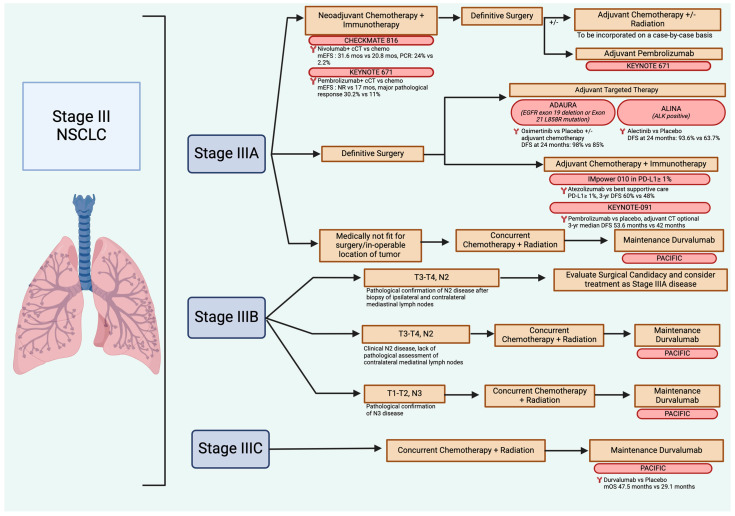
A summary of the FDA approved treatment approaches to the perioperative management of Stage III non-small cell lung cancer. Created with BioRender.com.

Patients with stage IIIA NSCLC disease include those with T3/T4, N0/N1 disease and T1/T2, N2 disease (Figure 2). Clinicians evaluating patients with stage IIIA disease should assess whether the patients are medically optimal candidates for thoracic surgery, taking into account their overall health, comorbid conditions, and lung reserves. If patients are medically and surgically fit to undergo surgery, those with T3/T4, N0/N1 disease can be considered for neoadjuvant chemo-immunotherapy for three cycles with carboplatin/paclitaxel and nivolumab followed by response assessment [81]. Those with a clinical response can proceed to surgery followed by an option for adjuvant chemotherapy after a multidisciplinary discussion between surgery, medical oncology, and radiation oncology. Neoadjuvant chemo-immunotherapy followed by adjuvant pembrolizumab is also an option based on the KEYNOTE-671 data and is also part of standard-of-care recommendations [84].

Another approach is to treat these patients with surgery first, followed by adjuvant chemotherapy for four cycles and immunotherapy for 1 year in those with PD-L1 > 1%. The data from the Impower010 study shows that the majority of patients who benefit from this approach have non-squamous histologies and PD-L1 expression ≥50% [71]. Data from Keynote-091 support adjuvant pembrolizumab following adjuvant chemotherapy in those with a PD-L1 ≥ 1% [70].

Patients with T1/T2, N2 disease can also be considered for neoadjuvant chemo-immunotherapy followed by surgery if they do not have bulky mediastinal lymph nodes or multi-station mediastinal lymph node disease [101]. Patients with single-station N2 non-bulky disease are good candidates for neoadjuvant chemo-immunotherapy prior to definitive surgery.

Patients discovered to have activating EGFR mutations are candidates for 3 years of adjuvant osimertinib after surgical resection and adjuvant chemotherapy. There is no documented benefit of immunotherapy in these patients [95]. Similarly, adjuvant alectinib is the approach of choice for patients with an ALK mutated resectable NSCLC (Stage IB-IIIA) without adjuvant chemotherapy [100]. 

Patients with stage IIIA disease who are not good candidates for surgery due to underlying medical reasons, or multi-station or bulky mediastinal lymph nodes should be offered chemoradiation followed by immunotherapy. Patients who may require pneumonectomy if surgery is pursued due to the location of the tumor are also best treated with primary chemoradiation followed by immunotherapy, given the poor surgical outcomes associated with pneumonectomy [54].

### 12.2. Stage IIIB

Patients with stage IIIB disease include those with T3/T4, N2 disease and those with T1/T2, N3 disease. Patients with T3/T4, N2 disease are potential surgical candidates, provided that the primary tumor is surgically resectable without requiring pneumonectomy, they do not have multi-station N2 disease, there is adequate organ function, and they are medically fit to undergo surgery [102]. Provided they have no driver mutations (EGFR classical mutations, ALK mutations), these patients should be considered for neoadjuvant chemo-immunotherapy before surgery with consideration for adjuvant immunotherapy.

Patients with T1/T2, N3 disease and those with T3/T4, N2 disease are not suitable for surgery and should be offered chemoradiation followed by immunotherapy.

### 12.3. Stage IIIC

Patients with Stage IIIC (including T3/T4, N3) disease should be offered primary chemoradiation followed by immunotherapy or can be treated similar to patients with metastatic NSCLC. There is no role for surgery or neoadjuvant chemo-immunotherapy in these patients. A summary of the standard-of-care approach to the management of Stage III NSCLC is highlighted in Figure 2.

## 13. Conclusions

The treatment of patients with NSCLC has become increasingly complex as the treatment landscape continues to evolve. Given the complicated therapeutic landscape, a multidisciplinary team remains critical in the treatment of patients with NSCLC. Of utmost importance is an accurate tumor stage and lymph node classification, since prognosis and treatment decisions are heavily dependent on disease stage. For patients with stage IIIA disease and the small subset of those with stage IIIB, in whom surgery remains a treatment option, it is important to assess their surgical candidacy and risks, and to offer neoadjuvant chemo-immunotherapy or adjuvant chemotherapy, immunotherapy, or targeted therapy based on individual patient characteristics. For patients with unresectable stage III NSCLC, the mainstay of treatment is concurrent chemoradiotherapy followed by immunotherapy.

Additionally, with the development of targeted therapies and immunotherapy in early-stage lung cancer, knowledge about the potential adverse effects of these drugs and the ability to recognize them promptly is critical. As there are many ongoing clinical trials in stage III NSCLC, the treatment paradigm will continue to evolve, leading to more treatment options and better future outcomes for patients.

## Figures and Tables

**Table 1:** Eighth edition TNM staging.

T/M	Label	N0	N1	N2	N3
T1	T1a ≤ 1 cm	IA1	IIB	IIIA	IIIB
	T1b > 1–2 cm	IA2	IIB	IIIA	IIIB
	T1c > 2–3 cm	IA3	IIB	IIIA	IIIB
T2	T2a (central tumor, visceral pleura)	IB	IIB	IIIA	IIIB
	T2a > 3–4 cm	IB	IIB	IIIA	IIIB
	T2b > 4–5 cm	IIA	IIB	IIIA	IIIB
T3	T3 > 5–7 cm	IIB	IIIA	IIIB	IIIC
	T3 (invading chest wall, pericardium, phrenic nerve)	IIB	IIIA	IIIB	IIIC
	T3 (separate tumor nodule[s] in the same lobe)	IIB	IIIA	IIIB	IIIC
T4	T4 > 7 cm	IIIA	IIIA	IIIB	IIIC
	T4 (tumor invading: mediastinum, diaphragm, heart, great vessels, recurrent laryngeal nerve, carina, trachea, esophagus, spine)	IIIA	IIIA	IIIB	IIIC
	T4 (tumor nodule in a different ipsilateral lobe)	IIIA	IIIA	IIIB	IIIC
M	M1a (contralateral nodes)	IVA	IVA	IVA	IVA
	M1a (pleural/pericardial dissemination)	IVA	IVA	IVA	IVA
	M1b (single extrathoracic metastasis)	IVA	IVA	IVA	IVA
	M1c (multiple extrathoracic metastasis)	IVB	IVB	IVB	IVB

Adapted from the eighth edition lung cancer stage classification. *Chest Journal* 2017;(151:1):193–203 [18]. Stage III is highlighted in green. Nodal stations for lung cancer include: N0—no lymph node involvement; N1—stations 10–14; N2—ipsilateral mediastinal lymph nodes; N3—contralateral hilar/mediastinal or supra-clavicular.

**Table 2:** Phase II and phase III trials evaluating the incorporation of immunotherapy with chemoradiation in stage III NSCLC. cCT, concurrent chemotherapy; cCRT, concurrent chemoradiotherapy.

Trial Name	Study Phase	Experimental Arm	Control Arm	Clinical Trials Identifier
PACIFIC-2 [65]	Phase 3	cCRT followed by durvalumab	cCRT followed by placebo	NCT03519971
PACIFIC-5 [66]	Phase 3	cCRT followed by fixed-dose durvalumab	cCRT followed by placebo	NCT03706690
COAST [62]	Phase 2	Arm 1: cCRT followed by durvalumab Arm 2: cCRT followed by durvalumab + oleclumab Arm 3: cCRT followed by durvalumab + monalizumab	-	NCT03822351
Alliance Foundation Study [64]	Phase 2	2 or 4 cycles of induction atezolizumab → cCRT → 2 cycles of consolidation chemotherapy with carboplatin and paclitaxel → adjuvant atezolizumab for 1 year of therapy from the start of induction.	-	NCT03102242
CONSIST	Phase 3	cCRT followed by sintilimab for 1 year	cCRT alone	NCT03884192
GEMSTONE-301 [67]	Phase 3	cCRT followed by Sugenlimab for 2 year	cCRT followed by placebo	NCT03728556
KEYNOTE-799 [68]	Phase 2	One cycle of chemotherapy with pembrolizumab → cCRT with 2 cycles of pembrolizumab → pembrolizumab × 14 cycles	-	NCT03631784
CheckMate 73L [69]	Phase 3	Arm 1: cCRT with nivolumab → nivolumab + ipilimumab Arm 2: cCRT with nivolumab → nivolumab	cCRT followed by durvalumab	NCT04026412

**Table 3:** Neoadjuvant and perioperative phase 2 and phase 3 trials evaluating the incorporation of immunotherapy to treat NSCLC.

Trial Name	Study Phase	Experimental Arm	Control Arm	Clinical Trials Identifier
KEYNOTE-671 [89]	Phase 3	Perioperative: Neoadjuvant Pembrolizumab + concurrent chemotherapy (cCT), adjuvant pembrolizumab	Neoadjuvant placebo + cCT, adjuvant placebo	NCT03425643
CHECKMATE-816 [81]	Phase 3	Neoadjuvant nivolumab + cCT	Neoadjuvant chemotherapy	NCT02998528
CHECKMATE-77T [90]	Phase 3	Neoadjuvant nivolumab + cCT, adjuvant nivolumab	Neoadjuvant placebo + cCT, adjuvant placebo	NCT04025879
AEGAN [91]	Phase 3	perioperative durvalumab + cCT	Neoadjuvant placebo + cCT	NCT04025879
Impower-030	Phase 3	Neoadjuvant atezolizumab + cCT, adjuvant atezolizumab	Neoadjuvant placebo + cCT	NCT03456063
NA_00092076	Phase 2	Exp Arm b: Neoadjuvant nivolumab + cCT Exp Arm c: Neoadjuvant nivolumab		NCT02259621
LCMC3 [92]	Phase 2	Neoadjuvant atezolizumab, adjuvant atezolizumab		NCT02927301
NEOSTAR [84]	Phase 2	Exp Arm A: Neoadjuvant nivolumab Exp Arm B: Neoadjuvant nivolumab + ipilimumab Exp Arm C: Neoadjuvant nivoumab + cCT		NCT03158129
NADIM [93]	Phase 2	Neoadjuvant nivolumab + cCT, adjuvant nivolumab		NCT03081689
NADIM II [82]	Phase 2	Neoadjuvant nivolumab + cCT, adjuvant nivolumab	Neoadjuvant chemotherapy	NCT03838159
IONESCO [80]	Phase 2	Neoadjuvant durvalumab		NCT03030131
SAKK 16/14 [94]	Phase 2	Sequential neoadjuvant chemotherapy and durvalumab, adjuvant durvalumab		NCT02572843
PRINCEPS [80]	Phase 2	Neoadjuvant atezolizumab		NCT02994576
